# Foliar iron application delays flavonoid degradation during the later grain-filling stage and regulates sugar metabolism in colored rice

**DOI:** 10.3389/fpls.2026.1787767

**Published:** 2026-04-07

**Authors:** Xia Zhao, Weiwei Wang, Yurou Jiang, Linghui Wang, Xinbin Liu, Hongyu Xie

**Affiliations:** 1 Faculty of Agriculture, Forestry and Food Engineering, Yibin University, Yibin, China; 2 Key Lab of Aromatic Plant Resource Exploitation and Utilization in Sichuan Higher Education, Yibin University, Yibin, China; 3 Solid-State Fermentation Resource Utilization Key Laboratory of Sichuan Province, Yibin University, Yibin, China

**Keywords:** colored rice, flavonoids, foliar iron fertilizer, later filling stage, sugar metabolism

## Abstract

**Introduction:**

Colored rice is recognized for nutritious function due to its flavonoid compounds. Flavonoid aglycones are conjugated with various sugar moieties. Iron (Fe) is an essential mineral nutrient for plants; however, the regulatory mechanisms by which Fe influences flavonoid accumulation dynamics and sugar metabolism remain unclear.

**Methods:**

FeSO_4_ was foliar-applied to black rice cultivar Nanheinuo and red rice cultivar Yuhongdao 5815 to study regulatory mechanisms by which Fe influences flavonoid accumulation dynamics and sugar metabolism.

**Results and discussion:**

The content of anthocyanins, proanthocyanidins, and total flavonoids in the grains decreased throughout the later filling stages. Fe application delayed their degradation during this phase. The differential expression of numerous genes associated with flavonoid transformation may be the primary reason. Genes annotated as key enzymes involved in phenylpropanoid and flavonoid biosynthesis were significantly upregulated in Nanheinuo. Additionally, foliar Fe application significantly impacted sugar and sugar alcohol content by regulating the expression of genes involved in polysaccharide decomposition, phosphorylation and dephosphorylation processes, and sugar interconversion. A close relationship was observed between sugar content and flavonoid-related compound content in the grains of colored rice. Multiple hub genes annotated as glycoside hydrolases in KEGG occupied central nodes within the co-expression network, suggesting that glycoside hydrolases may mediate the crosstalk between sugar and flavonoid metabolism in colored rice grains under Fe treatment, although glycosyltransferases primarily function in flavonoid glycosylation. Foliar Fe application delays flavonoid degradation during the later filling stage and regulates sugar metabolism in colored rice, thereby influencing flavonoid enrichment and eating quality.

## 1 Introduction

The colors of colored rice grains are primarily red and black. Anthocyanin accumulation results in black kernels, while proanthocyanidin accumulation leads to red kernels (Wongsa, 2020; Zhu et al., 2024). Understanding the dynamic accumulation patterns and regulatory mechanisms of flavonoids throughout the development of red and black rice grains is crucial for improving the nutritional quality of colored rice.

Flavonoids encompass several subcategories, including isoflavones, flavones, flavanones, proanthocyanidins, flavonols, and anthocyanins. All of these compounds are synthesized via the phenylpropanoid metabolic pathway (Gao et al., 2022). They have been extensively characterized in terms of molecular structure, genetic regulation, biosynthetic pathways, and the enzymes involved. The key enzymes involved in flavonoid metabolism primarily include phenylalanine ammonia lyase (PAL), coumaroyl-CoA ligase (4CL), chalcone isomerase (CHI), flavonoid 3’,5’-hydroxylase (F3’5’H), flavonoid 3’-monooxygenase (F3’H), chalcone synthase (CHS), naringenin 3-dioxygenase (F3H), dihydroflavonol 4-reductase (DFR), anthocyanin synthase (ANS), and UDP-glucosyltransferase (UGT) (Nayeem et al., 2021; Naik et al., 2022; Chen et al., 2023). Flavonoids play vital roles in plant growth, development, responses to abiotic stress and defense mechanisms (Jayaraman et al., 2021; Lahari et al., 2024). Numerous studies have focused on increasing flavonoid enrichment (Yamuangmorn et al., 2018; Jayaraman et al., 2021; Lahari et al., 2024; Xu et al., 2025). Iron (Fe) is an essential mineral nutrient for plants, contributing to various physiological processes. Fe availability significantly influences plant metabolism and the enrichment of nutrient compounds (Wang et al., 2021). As a cofactor, Fe markedly affects the activity of enzymes involved in the flavonoid biosynthesis, including monooxygenases such as F3’5’H and F3’H; dioxygenases such as ANS and F3H; as well as peroxidase (POD) and polyphenol oxidase (PPO) (Zeb et al., 2019; Wei et al., 2021; Liu et al., 2022). Shi et al. (2018) revealed that foliar Fe application increased anthocyanin and flavonoid content in grape berries. Similarly, He et al. (2021) reported comparable results in red rice, showing that the upregulated expression of F3H and ANS genes supported the increase in proanthocyanidin content. Our previous research also revealed that foliar Fe spraying promoted flavonoid metabolism in colored rice at the mature stage (Zhao et al., 2024). Despite the confirmed facilitating effect of foliar Fe fertilizer treatment on flavonoid metabolism has been confirmed in colored rice, the detailed mechanisms underlying Fe-induced flavonoid accumulation in colored rice grains remain to be fully elucidated.

Flavonoid aglycones are commonly conjugated with various sugar moieties, which influence the properties of flavonoids, including their stability, solubility, and biological activity. These properties are critical for the storage and transport of flavonoids within plant cells (Chen et al., 2024; Ren et al., 2022). The diversity of the medicinal functions and antioxidant ability of flavonoids can be improved by modifications in glycosylation (Zhan et al., 2019; Zagrean-Tuza et al., 2020). The sugar moieties of flavonoids contain glucosides, rhamnosides, galactosides, arabinosides, and rutinosides, suggesting potential crosstalk between sugar and flavonoid metabolism. Glycosylation is catalyzed by transglycosidases (TGs), glycoside hydrolases (GHs), and glycosyltransferases (GTs), with GTs primarily responsible for catalyzing glycosylation (Luang et al., 2013; Lai et al., 2025). After flowering, flavonoids begin to be transported to the rice caryopsis. The dry matter of the caryopsis increases rapidly during the early stages and then keeps relatively constant during later stages. The primary component of dry matter is starch (Guo et al., 2024). Sucrose, the main product of photosynthesis, is transported over long distances from the leaves to the caryopsis. Within the caryopsis, sucrose is primarily hydrolyzed into glucose and fructose by acid invertase (Braun et al., 2014; Jiang et al., 2020). Glucose and fructose are then used to synthesize starch in the endosperm or converted into other sugars for the synthesis of various substances (Liu et al., 2024). Hence, sugar metabolism is highly active in the caryopsis throughout the filling stage (Ma et al., 2017; Fei et al., 2021). Sugar accumulation in grains is an important quality factor, as it not only influences its nutritional value but also affects the flavor of rice by contributing sweetness and serving as substrates for the Maillard reaction, which results in the aroma of rice (Guan and Zhang, 2022). However, the effect of iron on sugar metabolism remains unclear.

Numerous studies have independently investigated sugar and flavonoid metabolism in rice (Ma et al., 2017; Fei et al., 2021; He et al., 2021). The relationship between these two metabolic pathways has also been observed in tea (Lv et al., 2022), embryo axes of yellow lupine (Morkunas et al., 2011), and fungi (Jia et al., 2025). However, crosstalk between sugar and flavonoid metabolism has not been reported in the grains of colored rice. Furthermore, the regulatory mechanisms of Fe in sugar metabolism and flavonoid accumulation dynamics in colored rice grains remain poorly understood. In this study, we aimed to elucidate these mechanisms. The findings will contribute to enhancing flavonoid enrichment in colored rice and improving rice quality.

## 2 Materials and methods

### 2.1 Plant materials and experiment design

The experimental field was located at the experimental farm of Yibin University in Yibin City, Sichuan Province, China. All field experiments were conducted in 2023 and 2024. Paddy soil was used for this study, with a pH of 5.92, 30.28 mg kg^-1^ available iron, 96.41 mg kg^-1^ available nitrogen, 70.35 mg kg^-1^ exchangeable potassium, and 27.63 mg kg^-1^ available phosphorus. The black rice variety Nanheinuo (BR) and the red rice variety Yuhongdao 5815 (RR) were used in this research. The seeds were sown in seedling trays and cultivated for approximately 27 days. The seedlings were transplanted into 20 L pails and cultivated under natural environmental conditions. The average temperature at the experimental site ranged from approximately 23 °C to 28 °C, characteristic of a subtropical humid monsoon climate. A completely randomized design with three biological replicates was implemented (Liu and Ming, 2020). And a biological replicate contained six pails. A compound fertilizer (2 g) was applied to each pail as a base fertilizer before transplantation. Additionally, 2 g of urea was applied at both the tillering and jointing stages.

Based on the results of the preliminary experiment for screening Fe concentration, the foliar Fe treatments were applied as follows: (1) Control, using deionized water as the foliar spray; (2) 1Fe, with 0.1% FeSO_4_·7H_2_O (w/v) as the foliar fertilizer; and (3) 2Fe, with 0.2% FeSO_4_·7H_2_O (w/v) as the foliar fertilizer. All foliar solutions contained Fe absorption enhancer nicotinamide (0.5% w/v) and surfactant Tween 80 (0.005% v/v). Approximately 40 mL of solution was applied to the leaves of the plants in each bucket. Foliar spraying was conducted after sunset at the end of the booting stage and repeated 7 d later. Panicles of BR and RR were sampled at 10, 15, 20, 25, 30, 35, and 40 days after flowering (DAF). Samples were rapidly frozen in liquid nitrogen and then stored at −80 °C for following analyses. The rest of rice panicles were harvested at maturity.

### 2.2 Content of proanthocyanidins, total flavonoids, and total phenolics, and antioxidant capacity

#### 2.2.1 Extract preparation

The panicles of BR and RR were sampled at 10, 15, 20, 25, 30, 35, and 40 DAF, with three biological replicates. Superior grains were selected and hulled. Approximately 0.2 g of hulled grains were ground into a homogenate using 10 mL of an extracting solution containing 75% acetone and 0.5% acetic acid. Sonication was then performed at ambient temperature for 35 min. All samples were shaken for 1 h at 25 °C, subsequently, centrifuged at 9,000 × g for 12 min. The supernatants were conserved for measurement. The extraction process was conducted under dark condition (Prior et al., 2010).

#### 2.2.2 Measurement of proanthocyanidins, total flavonoids, and total phenolics

Measurement of proanthocyanidin content was performed by the cinnamyl aldehyde assay method (Prior et al., 2010). In this method, 1.4 mL of blank, standard (catechin), or sample extracts were mixed with 4.2 mL of cinnamyl aldehyde solution, which contained 0.1% cinnamyl aldehyde reagent, 4.5% hydrochloric acid, and 68% ethanol. The mixtures were reacted in a 35 °C for 25 min. Finally, the value of absorbance was determined at 640 nm. The proanthocyanidin content of the samples was defined as milligrams of catechin per gram of sample.

The determination of total flavonoid content was performed according to the method described by Shen et al. (2009). The total flavonoid content was quantified based on a rutin standard curve. Total phenolic content was detected using a modified Folin-Ciocalteu reagent method and calculated using a gallic acid standard curve (Shen et al., 2009).

#### 2.2.3 Measurement of antioxidant capacity

The radical scavenging activities of 2,2’-azinobis-(3-ethylbenzothiazoline-6-sulfonate) (ABTS) and 1,1-diphenyl-2-picrylhydrazyl (DPPH) were measured following the protocols described by Jeng et al. (2010) and Shen et al. (2009), respectively. Ascorbic acid was used to construct the standard curves for both ABTS and DPPH assays. The radical scavenging activities of DPPH and ABTS were expressed as ascorbic acid equivalents per unit dry weight.

### 2.3 Measurement of anthocyanin content

Approximately 0.3 g of each sample, collected at different developmental stages, was immersed in 12 mL of acidified ethanol containing 0.2% HCl and 94% ethanol, then shaken at 25 °C for 19 h. The mixture was centrifuged at 9,000 × g for 6 min to obtain the supernatant, which was then utilized for anthocyanin analysis at 510 nm with a spectrophotometer. Anthocyanin content was quantified based on a standard curve of cyanidin-3-glucoside chloride.

### 2.4 Enzyme activity

The hulled grains collected 10 and 15 DAF were ground into a homogenate with an extraction solution. PAL and acid invertase activities were then measured using acid invertase and PAL colorimetric assay kits, respectively (Beijing, solarbio Technology, Co.,Ltd, Beijing, China).

### 2.5 Measurement of sugars and sugar alcohols

Freeze-dried grains at the mature stage were ground by a mixer mill. Then, 500 μL of an extraction solution containing water, isopropanol, and methanol (2:3:3, v/v/v) was sufficiently mixed with 20 mg of the resulting powder, subsequently, ultrasound treatment was applicated to the mixture for 30 min. The mixture was centrifuged at 13,000 × g (4 °C) for 7 min to obtain supernatant. 50 μL of the resulting supernatant was mixed with 20 μL of internal standard (1000 μg mL^-1^). The mixture was evaporated under a stream of nitrogen gas and then freeze-dried. Subsequently, derivatization was performed on the residue. Finally, the mixture was diluted to a suitable concentration for GC-MS analysis of sugars and sugar alcohols.

Agilent 8890 gas chromatograph coupled with a 5977B mass spectrometer, equipped with a DB-5MS column, was employed to GC-MS analysis. Standard solutions of sugars and sugar alcohols were prepared at various concentrations (0.001, 0.002, 0.005, 0.01, 0.02, 0.05, 0.1, 0.2, 0.5, 1, 2, 5, 10, 20, and 50 μg mL^-1^) to obtain quantitative peak intensity data for each concentration. Standard curves for different compounds were plotted using the ratio of their corresponding peak areas as the y-axis and the ratio of external standard to internal standard concentrations as the x-axis. The linear equations and determination coefficients of the standard curves for the substances detected in this study are presented in **Supplementary Table 1**.

Finely powdered rice samples at the mature stage were used to measure starch content with a colorimetric assay kit (Beijing Solarbio Technology Co., Ltd., Beijing, China).

### 2.6 Transcriptomic analysis

The grains at veraison, 10 DAF for BR and 15 DAF for RR, were collected for transcriptomic analysis, which was conducted by Biomarker Biotechnology Co., Ltd. (Beijing, China). Following PCR amplification and sequencing using the Illumina HiSeq™ 2500 platform, cDNA library construction and quality control were conducted. To obtain mapping information of the reads on the reference genome, the clean reads were aligned to the reference genome sequence (Oryza_sativa.IRGSP_1.0_20230606.genome.fa) using HISAT2 and subsequently assembled with StringTie. The mapping rates of reads ranged from 83.55% to 93.16%. Genes with an adjusted P-value < 0.01 and a fold change ≥ 1.5, as identified by DESeq2, were considered differentially expressed genes (DEGs). KEGG annotation was performed to characterize the functions of DEGs.

### 2.7 Quantitative real-time PCR analysis

Approximately 100 mg of grains were used to extract total RNA according to instruction provided with the RNAprep Pure Plant Kit (DP441) (TianGen Biotechnology Co., Ltd., Beijing, China). EasyScript One-Step gDNA Removal and cDNA Synthesis SuperMix was used to synthesize First-strand cDNA (TransGen Biotechnology Co.,Ltd, Co., Ltd., China). Specific primers of DEGs from transcriptome were designed by Primer 5 (**Supplementary Table 2**). Applied Biosystems™ Step StepOnePlus™ PCR System was conducted for Quantitative real-time PCR (qPCR) cycling with three technical replicates. The 2^△△CT^ method was used to analyze the relative transcript levels in gene expression.

### 2.8 Co-expression network and bioinformatic analyses

According to the protocol of Lv et al. (2022), construction of the gene co-expression network was based on the DEGs between the 1Fe and control treatments, as well as data on sugars and sugar alcohols with relatively high content, along with anthocyanins, proanthocyanidins, total flavonoids, and total phenolics, using weighted gene co-expression network analysis (WGCNA). The colors of modules were identified using the Dynamic Tree Cut algorithm. Cytoscape software was utilized to visualize the co-expression networks within key modules, and hub genes were selected based on their betweenness centrality (BC) values.

### 2.9 Statistical analysis

Analysis of variance (ANOVA) was employed to analyze the means of Fe treatment and control within one stage and one variety, based on data from three biological replicates. Duncan’s multiple range test (P<0.05) was adopted in this research. Correlation analysis was conducted using Pearson’s method. All data analyses and figure generation were performed using SPSS 26 and OriginPro 2024, respectively.

## 3 Results

### 3.1 Flavonoid-related compound content, antioxidant capacity, and enzyme activity during the filling stage of colored rice

**Figure 1:**
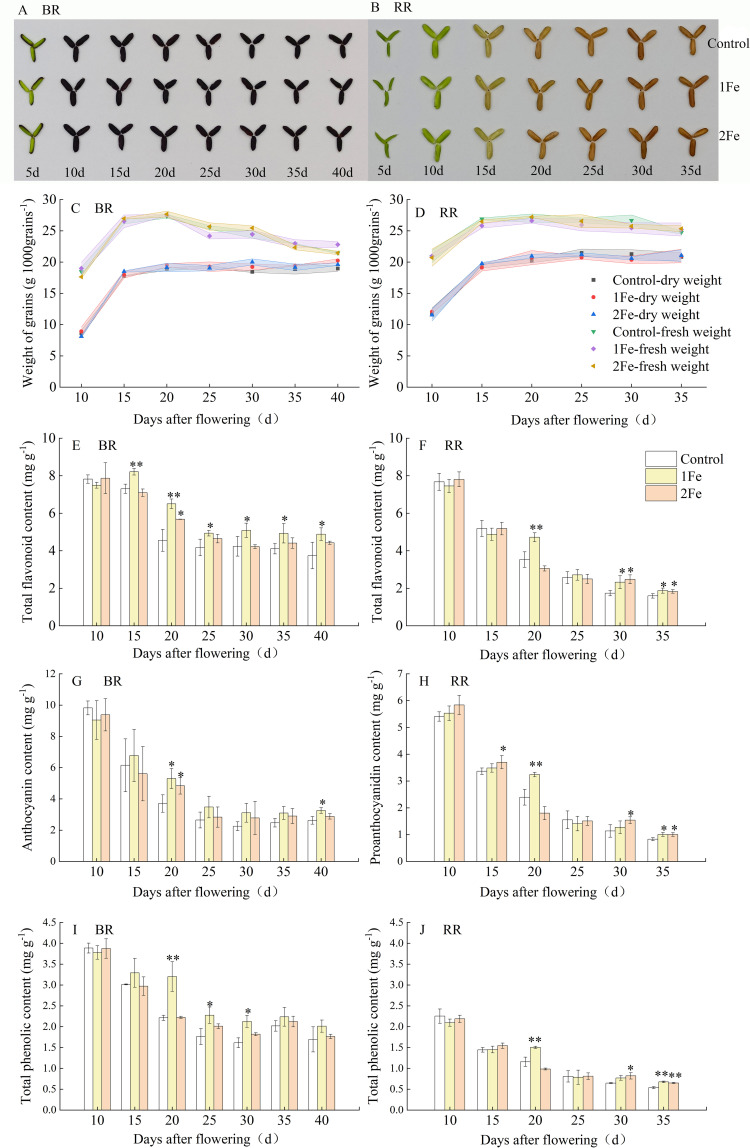
Changes in phenotype and flavonoid-related compounds of grains during the filling stage. Phenotypes at different stages of grain development in Nanheinuo (BR) **(A)** and Yuhongdao 5815 (RR) **(B)**. Changes in grain weight **(C, D)**, anthocyanin content in BR **(E)**, proanthocyanidin content in RR **(F)**, total flavonoid content **(G, H)**, and total phenolic content **(I, J)** during the filling stage. 1Fe: 0.1% (w/v) FeSO_4_·7H_2_O was sprayed onto the plants; 2Fe: 0.2% (w/v) FeSO_4_·7H_2_O was sprayed onto the plants. * and ** denote a significant difference at the 0.05 and 0.01 significance level according to Duncan's multiple range test, respectively.

**Figure 2:**
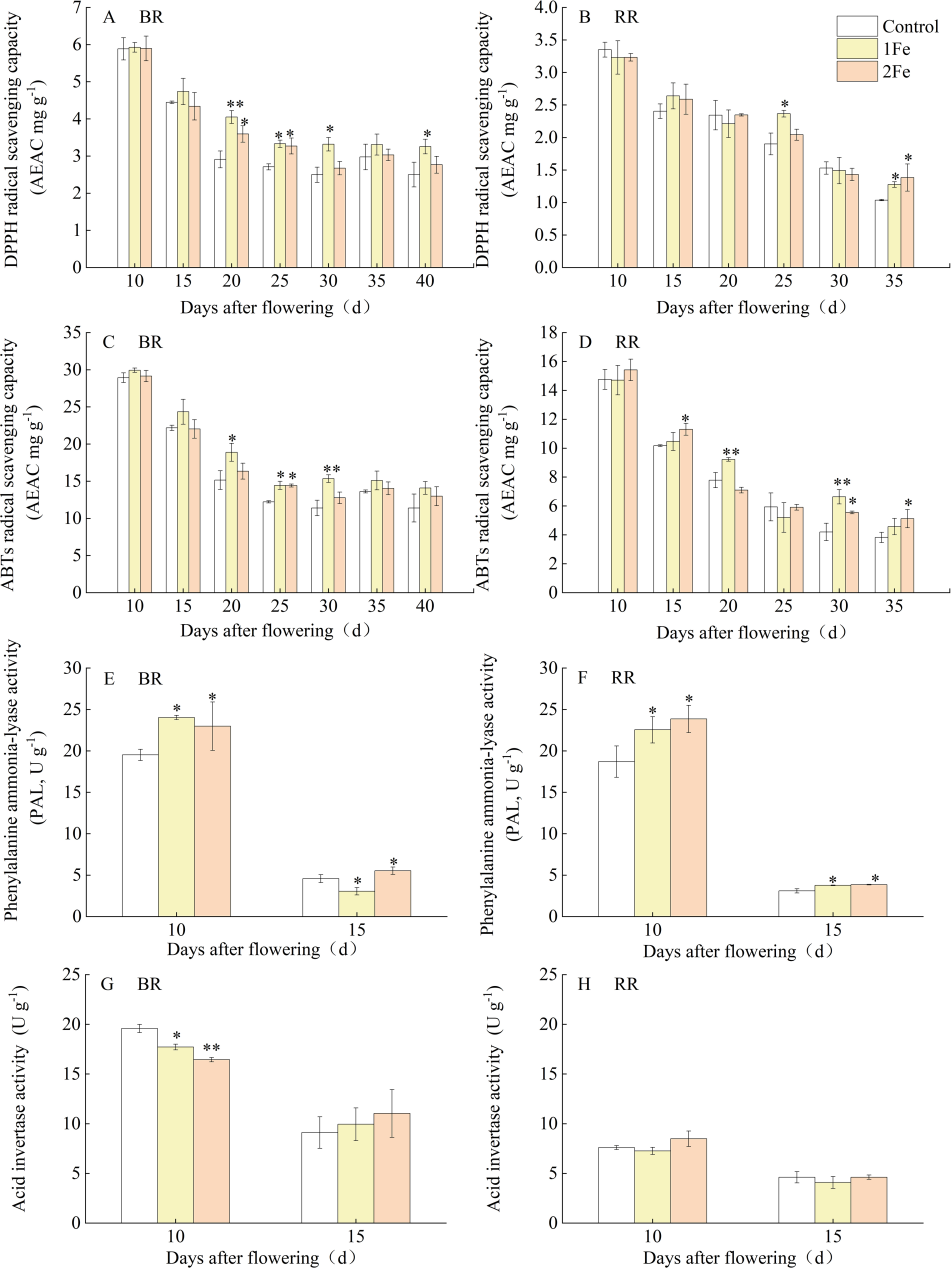
Changes in antioxidant capacity **(A–D)** and enzyme activity **(E–H)** of grains during the filling stage. AEAC, ascorbic acid equivalent antioxidant capacity. 1Fe: 0.1% (w/v) FeSO_4_·7H_2_O was sprayed onto the plants; 2Fe: 0.2% (w/v) FeSO_4_·7H_2_O was sprayed onto the plants. * and ** denote a significant difference at the 0.05 and 0.01 significance level according to Duncan's multiple range test, respectively.

To comprehensively and accurately characterize the accumulation dynamics of flavonoid-related compounds in colored rice grains, we collected grain samples at 10, 15, 20, 25, 35, and 40 days after flowering (DAF) from both BR and RR for measurement of anthocyanin, proanthocyanidin, total flavonoid, and total phenolic contents (**Figure 1**). The grain phenotypes at different filling stages for the two varieties are shown in **Figures 1A, B**. The entire BR grains turned black by 10 DAF, whereas the entire RR grains changed to green-yellow at 15 DAF and red at 20 DAF. Before 15 DAF, the dry matter in the grains accumulated rapidly. After this point, the dry matter weight remained relatively stable, while the fresh grain weight decreased, indicating that grains of both BR and RR began dehydrating after 15 DAF (**Figures 1C, D**). Anthocyanin, proanthocyanidin, total flavonoid, and total phenolic contents peaked at 10 DAF for both BR and RR. Subsequently, the levels of these compounds decreased throughout the later filling stages, and the extent of reduction in these compounds differed. The contents of these compounds in BR under the 1Fe treatment and in RR under both 1Fe and 2Fe treatments were significantly higher than those in the control treatment at multiple stages during the later grain-filling period (after 15 DAF). For BR, the contents of total flavonoids, anthocyanins, and total phenolics in the 1Fe treatment were significantly higher than those in the 2Fe treatment at certain stages, while no significant differences in these compound contents were observed between the 2Fe treatment and the control at multiple stages. (**Figures 1E–J**). The antioxidant capacity of two varieties, as measured by DPPH and ABTS scavenging rates, decreased during the later filling stage, corresponding with a decline in these compounds. Higher antioxidant capacity was also observed in the 1Fe and 2Fe treatments at several stages (after 15 DAF) compared to control. (**Figures 2A–D**). At the mature stage, Fe treatment significantly increased the total flavonoid content but did not significantly affect the DPPH and ABTS scavenging rates in BR. The activity of PAL, a key enzyme in flavonoid and phenolic biosynthesis, significantly decreased at 15 DAF compared to 10 DAF. Higher PAL activity under Fe treatment was also observed in BR at 10 DAF and in RR at both 10 and 15 DAF (**Figures 2E, F**).

### 3.2 Sugar metabolism under different treatments

**Figure 3:**
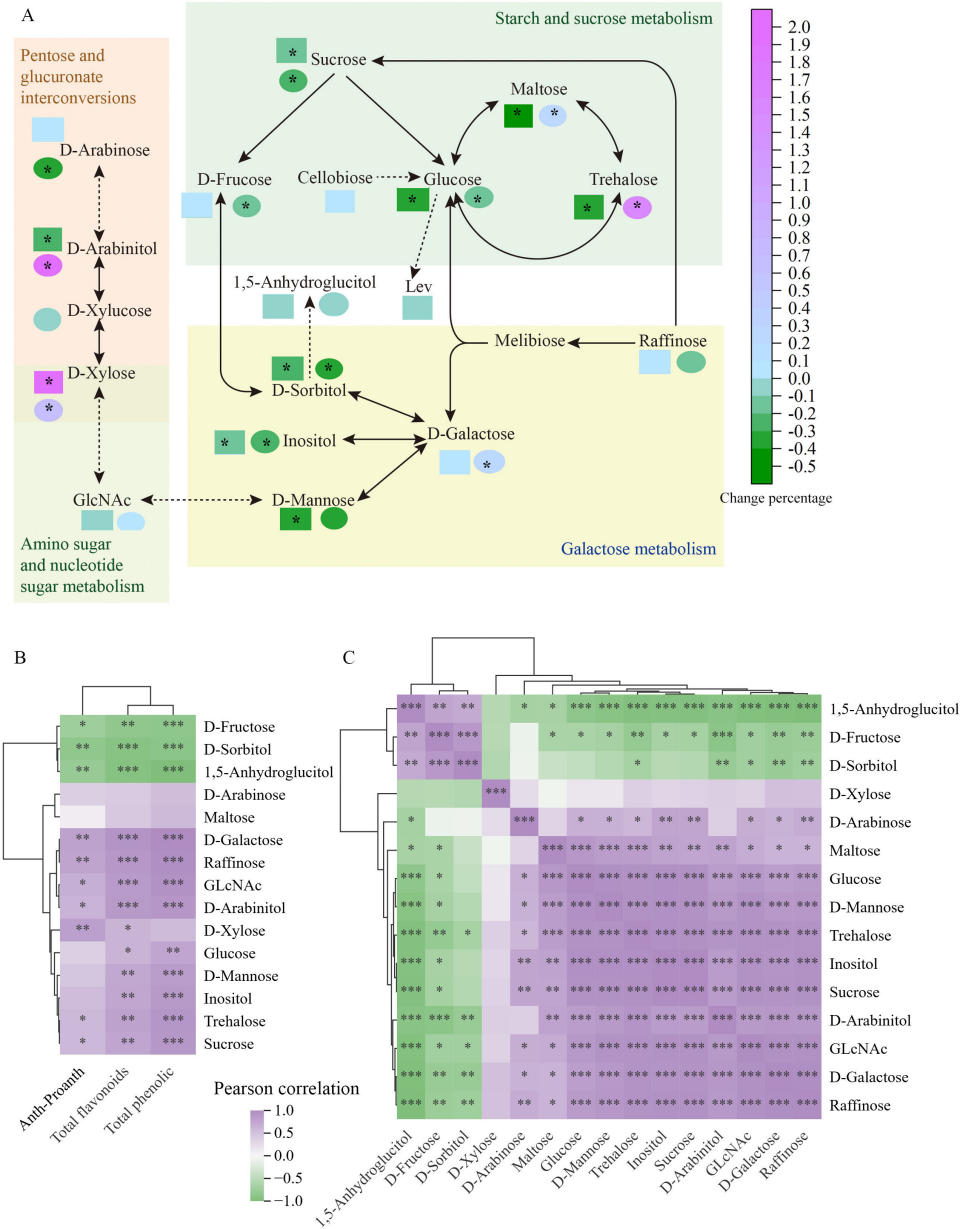
Effect of iron (Fe) treatment on sugar metabolism in the grains of colored rice **(A)**, Cluster correlation analysis chart between saccharides and flavonoid-related substances **(B)**, and cluster correlation analysis chart among saccharides **(C)**. The colors of the squares and circles represent the percentage increase or decrease compared to the control. Squares represent the response of Nanheinuo (BR) to Fe treatment, while circles indicate the response of Yuhongdao 5815 (RR) to Fe treatment. GLcNAc: 2-Acetamido-2-deoxy-D-glucopyranose. Lev: 1, 6-dehydrated -β -D-glucose. Anth-Proanth: anthocyanin content in BR and proanthocyanidin content in RR. Asterisk (*) in A denotes a significant difference at the 0.05 significance level according to Duncan’s multiple range test. *, **, and *** in **(B, C)** denote significant differences at the 0.05, 0.01, and 0.001 significance levels according to Pearson’s method, respectively.

The sugar and sugar alcohol content in grains is presented in **Supplementary Table 3**, including monosaccharides, disaccharides, trisaccharides, and polysaccharides. Foliar Fe fertilizer treatment prominently altered the sugar and sugar alcohol content in both BR and RR. Sugars and sugar alcohols with significant differences were primarily involved in KEGG pathways such as starch and sucrose metabolism, galactose metabolism, pentose and glucuronate interconversions, and amino sugar and nucleotide sugar metabolism (**Figure 3A**). The 1Fe treatment resulted in significantly lower levels of glucose, sucrose, D-sorbitol, and inositol compared to control, whereas only the content of D-xylose was significantly increased under the 1Fe treatment in both BR and RR. In BR, the 1Fe treatment significantly suppressed the accumulation of sugars and sugar alcohols, except for D-xylose, while the activity of acid invertase also suppressed under Fe treatment. In RR, the 1Fe treatment significantly raised the content of D-galactose, D-arabinitol, maltose, and trehalose, while significantly decreasing the content of D-fructose and D-arabinose compared to control. BR contained higher levels of most sugars and sugar alcohols compared to RR, and the activity of acid invertase in BR was also obviously higher than that in RR (**Figures 2G, H**). Foliar Fe treatment did not noticeably influence starch content (**Supplementary Table 3**).

### 3.3 Correlation between flavonoid and sugar metabolism

Foliar iron application simultaneously influences the deposition dynamics of flavonoid-related compounds and sugar metabolism. The correlations between the content of flavonoid-related compounds at 35 DAF in RR and at 40 DAF in BR, and the levels of sugars and sugar alcohols, are analyzed in **Figure 3B**. Anthocyanin content in BR and proanthocyanidin content in RR (Anth-Proanth) were significantly positively correlated with the levels of D-xylose, D-arabinitol, D-galactose, GlcNAc, sucrose, trehalose, and raffinose. Both total flavonoid and total phenolic contents were also strongly positively correlated with these sugar compounds, as well as with glucose, D-mannose, and inositol. Anth-Proanth, total flavonoid, and total phenolic contents were significantly negatively correlated with D-fructose, D-sorbitol, and 1,5-anhydroglucitol. Close relationships among sugars and sugar alcohols were also observed, and they were classified into three clusters based on their correlations (**Figure 3C**). D-fructose, D-sorbitol, and 1,5-anhydroglucitol formed one cluster, which negatively correlated with other sugar compounds except for D-xylose. D-xylose constituted a second cluster and showed little correlation with all other sugar compounds. The third cluster included D-arabinose, D-maltose, D-glucose, D-mannose, trehalose, inositol, sucrose, D-arabinitol, GlcNAc, D-galactose, and raffinose.

### 3.4 Global expression profiles

**Figure 4:**
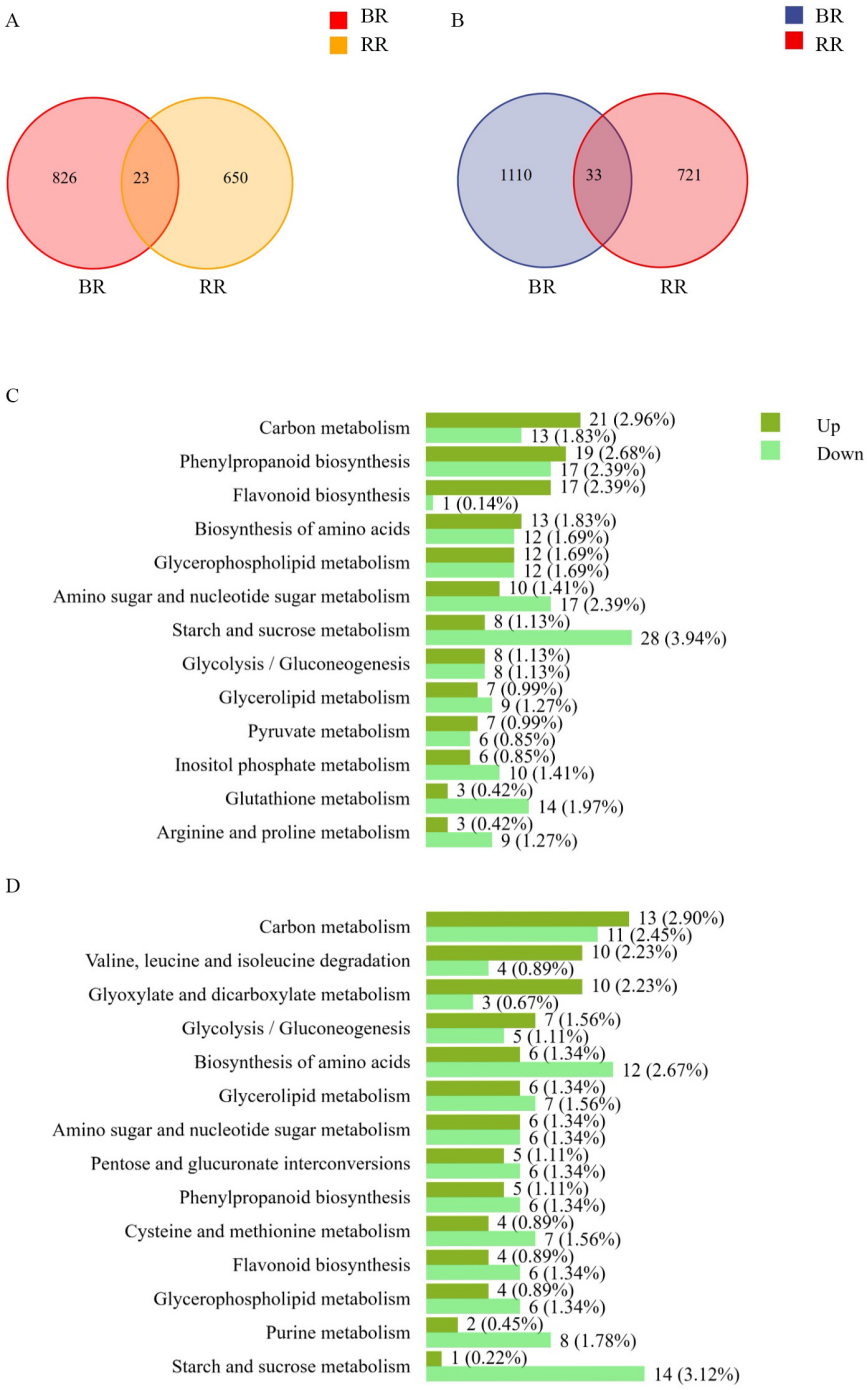
Summary of transcriptome datasets. Venn diagrams of up-regulated DEGs **(A)** and down-regulated DEGs **(B)**. Metabolic pathways of DEGs assigned to the KEGG database between the iron treatment and the control in black rice Nanheinuo (BR) **(C)** and red rice Yuhongdao 5815 (RR) **(D)**, DEGs: significantly differentially expressed genes.

To achieve systematic insights into the effects of foliar Fe treatment on the deposition mechanisms of flavonoids and related metabolic pathways, samples at veraison were collected for transcriptome analysis. **Supplementary Figure 1** shows a strong correlation between the transcriptomic data and the corresponding qPCR results (R² = 0.843), demonstrating that the transcriptomic dataset accurately represents transcript abundances. The heatmap and volcano plot of DEGs in BR and RR are presented in **Supplementary Figure 2**. Foliar iron application obviously influences the transcriptomic profiles of grains in colored rice. Both upregulated and downregulated DEG counts showed only a small number of genes simultaneously presented in both RR and BR (**Figures 4A, B**). Many DEGs were unique to either BR or RR, revealing differences in the regulatory mechanisms of Fe between the two varieties. Additionally, the primary KEGG metabolic pathways enriched with DEGs (with DEG counts ≥10) are shown in **Figures 4C, D**. In BR, many DEGs were involved in flavonoid biosynthesis and phenylpropanoid biosynthesis, while numerous DEGs were also associated with carbon metabolism, starch and sucrose metabolism, glycolysis/gluconeogenesis, and amino sugar and nucleotide sugar metabolism. A similar trend was observed in RR, where metabolic pathways related to flavonoids, including flavonoid biosynthesis and phenylpropanoid biosynthesis, were enriched. Additionally, many DEGs in RR were involved in sugar-related metabolic pathway, including carbon metabolism, starch and sucrose metabolism, glycolysis/gluconeogenesis, pentose and glucuronate interconversions, and amino sugar and nucleotide sugar metabolism.

### 3.5 Sugar- and flavonoid-related metabolic pathway

**Figure 5:**
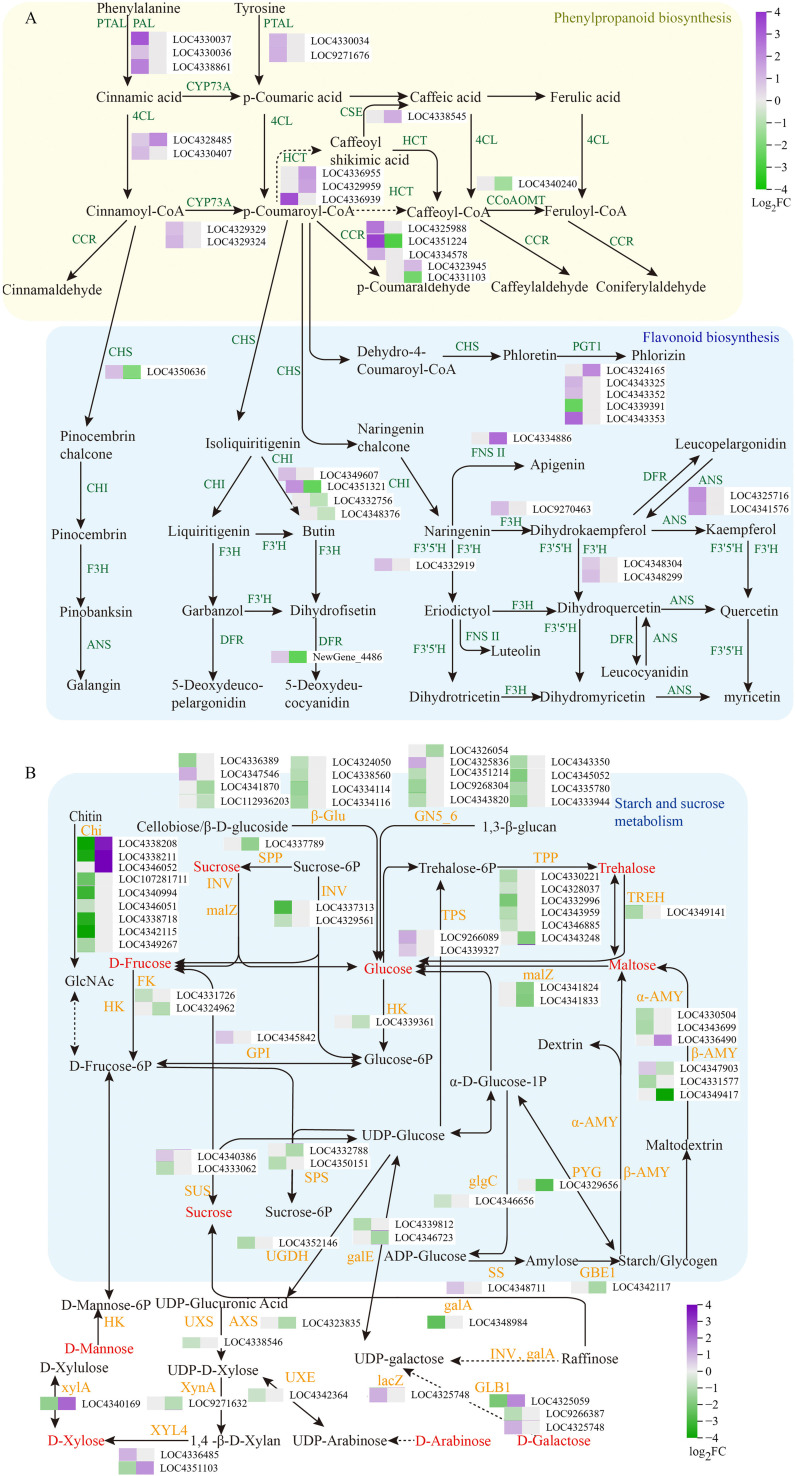
Analysis of flavonoid-related metabolic pathways **(A)** and sugar metabolism pathways **(B)**. FC denotes the fold change in gene expression compared to the control. The left squares display heat maps of differential gene expression in Nanheinuo (BR), while the right squares show heat maps of differential gene expression in Yuhongdao (RR). Grey indicates genes without significant differential expression. The sugars with significant differences are marked in red in figure **(B)** A heatmap of genes annotated as the same enzyme is presented only once. The enzymes in figure A are as follows: ANS, anthocyanin synthase; CHI, chalcone isomerase; F3’H, flavonoid 3’-monooxygenase; F3H, naringenin 3-dioxygenase; CHS, chalcone synthase; DFR, dihydroflavonol 4-reductase; 4CL, coumaroyl-CoA ligase; F3’5’H, flavonoid 3’,5’-hydroxylase; PTAL, phenylalanine/tyrosine ammonia-lyase; CYP73A, trans-cinnamate 4-monooxygenase; HCT, shikimate O-hydroxycinnamoyl-transferase; CCoAOMT, caffeoyl-CoA O-methyltransferase; CCR, cinnamoyl-CoA reductase; PGT1, phlorizin synthase; and FNS II, flavone synthase II. The enzymes in figure B include: INV, β-fructofuranosidase; FK, fructokinase; GPI, glucose-6-phosphate isomerase; glgC, glucose-1-phosphate adenylyltransferase; SS, starch synthase; β-AMY, β-amylase; α-AMY, α-amylase; TPS, trehalose 6-phosphate synthase; TPP, trehalose 6-phosphate phosphatase; SPS, sucrose-phosphate synthase; SUS, sucrose synthase; galE, UDP-glucose 4-epimerase; UGDH, UDP-glucose 6-dehydrogenase; UXS, UDP-glucuronate decarboxylase; UXE, UDP-arabinose 4-epimerase; XYL4, xylan 1,4-β-xylosidase; PYG, glycogen phosphorylase; malZ, α-glucosidase; HK, hexokinase; SPP, sucrose-6-phosphatase; β-Glu, β-glucosidase; GN5_6, glucan endo-1,3-β-glucosidase 5/6; GBE1, 1,4-α-glucan branching enzyme; XynA, 1,4-β-D-xylan synthase; GLB1, β-galactosidase; xylA, xylose isomerase; TREH, α-trehalase; lacZ, β-galactosidase; galA, α-galactosidase; and Chi, chitinase.

DEGs involved in sugar- and flavonoid-related metabolic pathways were further analyzed and are presented in **Figure 5**. The expression of genes annotated as key enzymes in phenylpropanoid and flavonoid biosynthesis, including ANS, CHI, F3’5’H, F3’H, CHS, 4CL, DFR, F3H, and PAL, was significantly upregulated in BR under Fe treatment. In contrast, both upregulation and downregulation of these DEGs were observed in RR. Additionally, multiple DEGs annotated as enzymes catalyzing transformations among various flavonoids were identified in both BR and RR. These include shikimate O-hydroxycinnamoyl-transferase (HCT), CHI, CHS, DFR, coumaroyl-CoA ligase (4CL), cinnamoyl-CoA reductase (CCR), and phlorizin synthase (PGT1). **Figure 5A** illustrates the specific reactions catalyzed by these enzymes.

Most genes annotated as enzymes catalyzing the decomposition of cellobiose, glucan, raffinose, sucrose, and starch/glycan showed significantly decreased expression levels under Fe treatment (**Figure 5B**). These enzymes include β-glucosidase (β-Glu), glucan endo-1,3-β-glucosidase 5/6 (GN5_6), β-fructofuranosidase (INV), β-amylase (β-AMY), α-amylase (α-AMY), glycogen phosphorylase (PYG), α-glucosidase (malZ), and α-galactosidase (galA). However, Fe treatment upregulated only four genes annotated as these enzymes. Genes annotated as enzymes involved in phosphorylation and dephosphorylation, including fructokinase (FK), hexokinase (HK), trehalose 6-phosphate phosphatase (TPP), and sucrose-6-phosphatase (SPP), were significantly downregulated under Fe treatment. Multiple DEGs annotated as enzymes catalyzing transformations among monosaccharides and disaccharides were identified. Fe treatment significantly downregulated the expression of multiple genes annotated as chitinase (Chi) in BR but upregulated them in RR.

### 3.6 Key modules and hub genes associated with sugar and flavonoid metabolism

**Figure 6:**
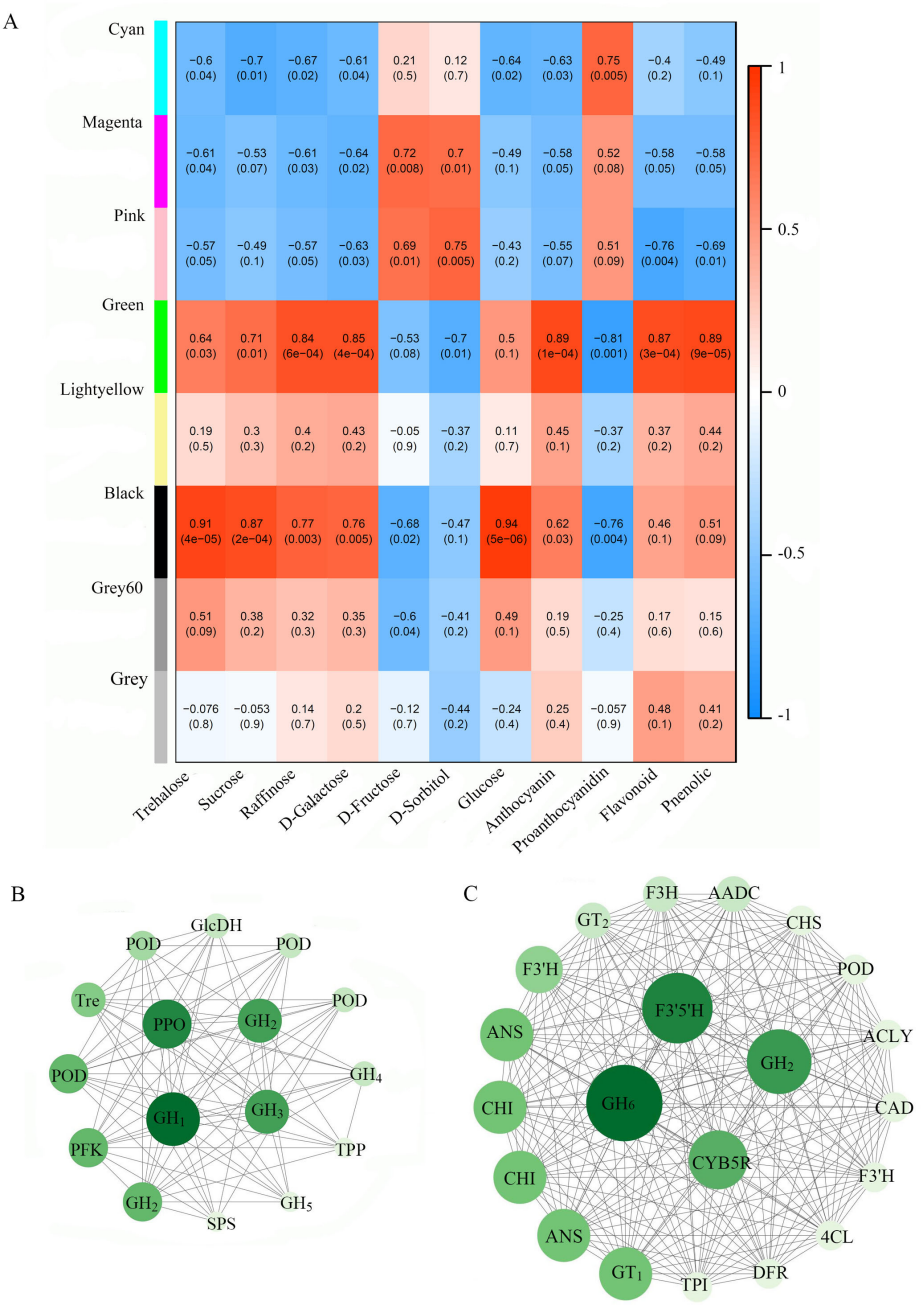
Correlations between sugar and flavonoid content and the expression levels of differentially expressed genes **(A)**. Network analysis of the black module **(B)** and the green module **(C)**. Notes: GH_4_, β-galactosidase; GH_5_, galacturan 1,4-α-galacturonidase; PFK, 6-phosphofructokinase 1; GH_3_, β-fructofuranosidase; POD, peroxidase; SPS, sucrose-phosphate synthase; Tre, trehalase; GH_1_, glucan endo-1,3-β-glucosidase; GH_2_, β-glucosidase; TPP, trehalose 6-phosphate phosphatase; GlcDH, UDP-glucose 6-dehydrogenase; PPO, polyphenol oxidase; GH_6_, β-D-xylosidase; F3’5’H, flavonoid 3’,5’-hydroxylase; CYB5R, cytochrome-b5 reductase; GT_1_, anthocyanin 3-O-glucosyltransferase; ANS, anthocyanin synthase; CHI, chalcone isomerase; F3’H, flavonoid 3’-monooxygenase; GT_2_, UDP-glycosyltransferase; F3H, naringenin 3-dioxygenase; AADC, aromatic L-amino acid decarboxylase; CHS, chalcone synthase; ACLY, ATP-citrate lyase; CAD, cinnamyl-alcohol dehydrogenase; DFR, dihydroflavonol 4-reductase; TPI, triosephosphate isomerase; 4CL, coumarin CoA ligase.

Based on DEGs, WGCNA was conducted to explore the metabolic mechanisms underlying the potential correlation between flavonoids and sugars. Key modules were identified through WGCNA and are represented by various colors (**Figure 6A**). Notably, the contents of sucrose, trehalose, raffinose, and galactose, along with anthocyanins, total flavonoids, and total phenolics, showed significant positive correlations with gene expression in the green module, with Pearson’s correlation coefficients exceeding 0.64. In contrast, the contents of sorbitol and proanthocyanidin were significantly negatively correlated with gene expression in the green module, with Pearson’s coefficients below -0.7. A similar pattern was observed in the black module. Specifically, the black module showed a strong positive correlation with glucose content (Pearson’s coefficient = 0.94) and strong negative correlations with fructose and proanthocyanidin contents. The black and green modules were selected for further analysis. KEGG annotation was performed to characterize the functions of genes within these modules. Co-expression networks were constructed using genes from the black and green modules. Hub genes associated with flavonoid and sugar metabolism were identified in the black and green modules, respectively. Co-expression networks were generated based on betweenness centrality (BC) values and are presented in **Figures 6B, C**. The black module contained sixteen hub genes, while the green module contained twenty-one. The hub genes from the black module included six genes annotated as GHs, four genes annotated as peroxidase (POD), one gene annotated as polyphenol oxidase (PPO), as well as genes annotated as 6-phosphofructokinase 1 (PFK), trehalose 6-phosphate phosphatase (TPP), trehalase (Tre), UDP-glucose 6-dehydrogenase (GlcDH), and sucrose-phosphate synthase (SPS) according to KEGG. The GHs and PPO genes occupied central nodes in the co-expression network. A cluster of hub genes annotated as key enzymes involved in flavonoid metabolism was identified in green modules, including ANS, CHI, F3’5’H, F3’H, CHS, 4CL, DFR, F3H, anthocyanin 3-O-glucosyltransferase (GT_1_), UDP-glycosyltransferase (GT_2_), and cinnamyl-alcohol dehydrogenase (CAD). However, two genes annotated as GHs also occupied central nodes within this co-expression network.

## 4 Discussion

### 4.1 Content of flavonoid-related compounds in the grains decreased throughout the later filling stages and was independent of dry matter accumulation

The biosynthesis and accumulation of flavonoid compounds occur during the grain-filling stage in colored rice (Shao et al., 2014; Rahman et al., 2015; Jiamyangyuen et al., 2017). In this study, dry matter in the grains accumulated rapidly before 15 DAF. After this point, dry matter weight remained relatively stable, while fresh grain weight decreased, indicating that grains of both BR and RR began dehydrating after 15 DAF (**Figures 1C, D**). Grain growth may influence the dynamic deposition of flavonoid compounds. Therefore, grain phenotype, total flavonoid, anthocyanin, proanthocyanidin, and total phenolic content were analyzed at different developmental stages. The entire BR grains turned black by 10 DAF, whereas the RR grains changed to green-yellow at 15 DAF and red at 20 DAF. The levels of all these compounds peaked at 10 DAF in both BR and RR. Subsequently, their concentrations decreased throughout the later filling stages. This phenomenon suggests that the accumulation and transformation of these compounds were independent of dry matter accumulation. Shao et al. (2014) and Rahman et al. (2015) also reported a significant decrease in anthocyanin content of black rice during the later filling stage. In contrast, Xiong et al. (2023) reported a black rice variety exhibiting slower accumulation of flavonoids and phenolics; the entire grain turned black at 21 DAF and reached the highest anthocyanin content at 28 DAF, without a subsequent decrease in anthocyanin levels. Different varieties of black rice had various anthocyanin accumulation dynamics (Zhao and Liu, 2021). The varietal discrepance may contribute to these contrasting results. The change in grain color of RR did not correspond directly with the decrease in proanthocyanidin and total flavonoid content during the filling stage, consistent with the results of Jiamyangyuen et al. (2017). This discrepancy might be due to the presence of colorless oligomeric proanthocyanidins during the early filling stage (Liao et al., 2025). The observed decline in anthocyanin, proanthocyanidin, and total flavonoid levels suggest that active metabolic transformations existed during the later filling stage. This decrease might be attributed to dehydration after 15 DAF and the reduced activity of PAL, a key enzyme involved in flavonoid biosynthesis (Chen et al., 2023).

### 4.2 Foliar iron application significantly influences the dynamics of flavonoid accumulation and sugar metabolism, while a close correlation between flavonoid and sugar metabolism exists in colored rice

Our previous studies reported that foliar Fe treatment increased the content of proanthocyanidins, anthocyanins, and total flavonoids, as well as the antioxidant capacity in mature grains of colored rice. However, it did not significantly affect the Fe content of the grains over a two-year experiment (Zhao et al., 2024). Thus, this study focused on the underlying regulatory mechanisms of flavonoid-related substances response to Fe treatment. Because the total flavonoid content of rice grains did not increase with rising Fe concentration and varieties responded differently to it (Zhao et al., 2024). Based on preliminary experiment results, 0.1% (1Fe) and 0.2% (2Fe) FeSO_4_·7H_2_O were selected as the optimal Fe concentrations for this study. For BR, the contents of total flavonoids, anthocyanins, and total phenolics in the 1Fe treatment were significantly higher than those in the 2Fe treatment at certain stages, while no significant differences in these compound contents were observed between the 2Fe treatment and the control at multiple stages. This phenomenon may be due to the different Fe dose responses. Nevertheless, the contents of these compounds in BR under the 1Fe treatment and in RR under both 1Fe and 2Fe treatments were significantly higher than those in the control treatment at multiple stages during the later grain-filling period, suggesting that Fe application enhances the accumulation of these compounds in mature grains primarily by retarding their degradation during this phase. Higher PAL activity under Fe treatment was also observed in BR at 10 DAF and in RR at both 10 and 15 DAF (**Figures 2E, F**), indicating that PAL activity might contribute to changes in total flavonoid content across different treatments. Although similar variation trends in flavonoid and anthocyanin contents were observed during the filling stage, and a strong correlation between them was confirmed (Zhu et al., 2024), the extent of reduction in these compounds differed due to the varying stability of different flavonoids during grain maturation. Additionally, variations in extraction and detection methods, as well as differences in error ranges, may have contributed to the inconsistent results in variance analysis between the Fe treatment and control groups. This phenomenon was also observed in the antioxidant capacity of the two varieties, despite the strong positive correlation between total flavonoid content and antioxidant capacity (Chen et al., 2024). Furthermore, different flavonoid metabolites exhibit varying antioxidant capacities (Chen et al., 2022). At the mature stage, Fe treatment significantly increased the total flavonoid content but did not significantly affect the DPPH and ABTS scavenging rates in BR. This suggests that Fe treatment may impact the transformation and storage forms of flavonoids during maturity. Our previous study reported foliar application of iron impacts flavonoid glycosylation and storage forms (Zhao et al., 2024). Overall, higher antioxidant capacity, DPPH and ABTS scavenging rates, were observed in the 1Fe and 2Fe treatments at several stages (after 15 DAF) compared to the control (**Figures 2A–D**). Although the differences in metabolite concentrations between the control and Fe treatments appear relatively small, they were consistently observed over many years of experimentation, with the magnitude of these differences varying annually (Zhao et al., 2024). This supports research on the effects of environmental factors on flavonoids and offers a new perspective for studying the nutritional physiology of trace elements in colored rice and the agronomic regulation of flavonoid compounds.

Flavonoid aglycones are commonly conjugated with various sugar moieties (Zhan et al., 2019). Additionally, sugar accumulation in grains is an important quality factor, as it not only influences nutritional value but also affects the flavor of rice by contributing sweetness and serving as substrates for the Maillard reaction, which results in the aroma of rice (Guan and Zhang, 2022). Therefore, sugar metabolism in grains, including monosaccharides, disaccharides, trisaccharides, and polysaccharides, were investigated. Foliar Fe fertilizer application resulted in significantly lower levels of glucose, sucrose, D-sorbitol, and inositol compared to control in both BR and RR, whereas only the content of D-xylose was prominently increased under the 1Fe treatment compared to control. Foliar Fe fertilizer application did not noticeably affect starch content (**Supplementary Table 3**). Sugars and sugar alcohols with significant differences were primarily involved in KEGG pathways such as starch and sucrose metabolism, galactose metabolism, pentose and glucuronate interconversions, and amino sugar and nucleotide sugar metabolism (**Figure 3**). Foliar iron application simultaneously influences the deposition dynamics of flavonoid-related compounds and sugar metabolism. Potential crosstalk between flavonoid and sugar metabolism has also been found in tea (Lv et al., 2022) and fungi (Jia et al., 2025). The correlations among these compounds in colored rice were investigated in this study. Anth-Proanth was significantly positively correlated with the levels of D-xylose, D-arabinitol, D-galactose, GlcNAc, sucrose, trehalose, and raffinose. Both total flavonoid and total phenolic contents were also strongly positively correlated with these sugar compounds, as well as with glucose, D-mannose, and inositol. Conversely, Anth-Proanth, total flavonoid, and total phenolic contents were significantly negatively correlated with D-fructose, D-sorbitol, and 1,5-anhydroglucitol (**Figure 3**). Farcuh et al. (2022) similarly reported that anthocyanin concentrations were positively associated with sucrose and galactose in plums. However, they found that flavonol and flavan-3-ol metabolism were positively correlated with sorbitol, fructose, glucose, and minor sugars in plums. This finding does not align with our results, which may be due to differences in measurement methods between total flavonoid content and the metabolism of flavonol and flavan-3-ol. Sugar moieties of flavonoids can influence their properties, including stability, solubility, and biological activity, all of which are crucial for the transport and storage of various flavonoids (Ren et al., 2022). The close relationship between sugar metabolism and the content of flavonoid-related compounds suggest that sugar metabolism may affect the dynamics of flavonoid transformation and accumulation in response to Fe treatments. Strong correlations among sugars and sugar alcohols were also observed. These correlational relationships and the transformations among these sugar compounds within metabolic pathways indicate adjustments in internal sugar metabolism, which may also influence flavonoid accumulation.

### 4.3 Foliar iron application influences the expression of genes involved in flavonoid and sugar metabolic pathway

Due to the active flavonoid metabolic network in the grains at veraison, the transcriptional characteristics during this period directly influences the accumulation and diversity of flavonoids, as well as the direction and efficiency of subsequent degradation and transformation processes. Simultaneously, physiological metabolism and enzymatic activity in the grains rapidly decline as they mature (Wang et al., 2021). This is demonstrated by the significantly greater number of down-regulated DEGs at 20 DAF compared to up-regulated genes, based on the analysis of gene expression differences between 15 DAF and 20 DAF (**Supplementary Figure 3**), and by the near-zero activity of PAL and acid invertase at 20 DAF measured in preliminary experiments. Low physiological activity contradicts the metabolic analysis of flavonoids and sugars. Therefore, transcriptome analysis and the qPCR validation of the grains at veraison (10 DAF for BR and 15 DAF for RR) are optimal for explaining the dynamic transformation of flavonoids during the later grain-filling stage. To obtain systematic insights into the effect of foliar Fe treatment on the deposition mechanisms of flavonoid and sugar metabolism, transcriptome analysis of the grains at veraison was carried out. It was observed that, whether considering upregulated or downregulated DEGs, only a small number of DEGs were common to both RR and BR. Man I thank the authors for the detailed response. However, I have a few additional queries. The revised abstract (lines 19-20) suggests that flavonoid transformation may explain delayed flavonoid degradation, and lines 284–285 mention DEGs associated with flavonoid transformation in BR and RR. Could the authors clearly specify what types of flavonoid transformations are being referred to and which genes or enzyme families are involved? Additionally, what are their potential roles in regulating flavonoid dynamics, and how has Fe treatment influenced their expression? Additional interpretations in result and discussion sections would help clarify the proposed role of Fe in regulating flavonoid transformation.y DEGs were unique to either BR or RR. These findings are consistent with the results of sugar metabolism described above. The responses of D-mannose, maltose, D-galactose, D-arabinitol, trehalose, D-fructose, and D-arabinose to Fe treatment differed between BR and RR. Variance in acid invertase activity further supported the differences in sugar metabolism between BR and RR. These results suggest distinct regulatory mechanisms of Fe in RR and BR, which may stem from the remarkable difference in flavonoid metabolism between the two colored rice types (Chen et al., 2012; Zhang et al., 2023; He et al., 2025). Nevertheless, DEGs were simultaneously enriched in KEGG pathways such as phenylpropanoid biosynthesis, flavonoid biosynthesis, carbon metabolism, starch and sucrose metabolism, amino sugar and nucleotide sugar metabolism, and glycolysis/gluconeogenesis in both BR and RR. Among these pathways, Fe treatment had a significant impact on the levels of multiple sugars and sugar alcohols involved in starch and sucrose metabolism, as well as amino sugar and nucleotide sugar metabolism, in both BR and RR (**Figure 3A**). Furthermore, DEGs involved in sugar- and flavonoid-related metabolic pathways were analyzed in greater detail. The expression of genes annotated as key enzymes in phenylpropanoid and flavonoid biosynthesis, including ANS, CHI, F3’5’H, F3’H, CHS, 4CL, DFR, F3H, and PAL, was significantly upregulated in BR under Fe treatment. This upregulation corresponded with the higher total flavonoid, total phenolic, and anthocyanin content observed in BR under Fe treatment. Additionally, multiple DEGs annotated as enzymes catalyzing transformations among various flavonoids were identified in both BR and RR, including shikimate O-hydroxycinnamoyl-transferase (HCT), CHI, CHS, DFR, coumaroyl-CoA ligase (4CL), cinnamoyl-CoA reductase (CCR), and phlorizin synthase (PGT1). **Figure 5A** illustrates the specific reactions catalyzed by these enzymes. Although the expression level changes of these DEGs in response to Fe treatment were different, this result further supports the conclusion that Fe treatment impacts the transformation and storage forms of flavonoids during maturation. The differential expression of numerous genes associated with flavonoid transformation may be a primary reason in delaying flavonoid degradation during the later filling stage, due to the varying stability of different flavonoids. However, this hypothesis requires further investigation.

Most genes annotated as enzymes responsible for the decomposition of cellobiose, glucan, raffinose, sucrose, and starch/glycan exhibited significantly decreased expression levels under Fe treatment, suggesting that Fe application might suppress polysaccharide degradation. Genes annotated as fructokinase (FK), hexokinase (HK), trehalose 6-phosphate phosphatase (TPP), and sucrose-6-phosphatase (SPP) were also significantly downregulated under Fe treatment. These enzymes involve in phosphorylation and dephosphorylation of sugar. Multiple DEGs annotated as enzymes that catalyze transformations among monosaccharides and disaccharides were identified, contributing to significant variance in the content of sugars and sugar alcohols between Fe treatment and control. Fe treatment significantly downregulated the expression of multiple genes annotated as chitinase (Chi) in BR but upregulated them in RR. Since Chi activity is closely correlated with biological resistance (Feng et al., 2025), the regulatory effect of Fe on biological resistance warrants further investigation. To explore the co-expression networks within gene modules strongly correlated with the content of sugar- and flavonoid-related substances, WGCNA was performed based on DEGs regulated by Fe application. Key modules (green and black) significantly correlated with most flavonoid compounds, sugars, and sugar alcohols, and contained multiple hub genes annotated as glycoside hydrolases (GHs) in KEGG, particularly in the black module, where these GH genes occupied central nodes within the co-expression network. GHs (EC 3.2.1.-), also known as glycosidases, catalyze the cleavage of glycosidic bonds in flavonoid glycosides. They can also utilize sucrose and starch as glycosyl donor substrates to catalyze O-glycosylation of flavonoids (Minic, 2008; Tian et al., 2024). Along with glycosyltransferases (GTs) and transglycosidases (TGs), GHs mediate the formation and cleavage of glycosidic linkages in flavonoid glycosides (Lai et al., 2025). However, GTs primarily drive flavonoid glycosylation and exhibit sugar-donor selectivity (Kim et al., 2013). In this study, only two hub genes annotated as anthocyanin 3-O-glucosyltransferase (GT1) and UDP-glycosyltransferase (GT2) were identified within the green module. These findings suggest that GHs and GTs connect sugar and flavonoid metabolism in the grains of colored rice under Fe treatment by regulating flavonoid glycosylation. The differential expression of multiple hub genes annotated as GHs, regulated by foliar Fe application, might mainly contribute the crosstalk between sugar and flavonoid metabolism in grains of colored rice. In addition to hub genes annotated as GHs and GTs, four hub genes annotated as peroxidase (POD) and one hub gene annotated as polyphenol oxidase (PPO) also warrant attention. As a cofactor, Fe markedly affects the activity of peroxidase (POD) and polyphenol oxidase (PPO) (Zeb et al., 2019; Wei et al., 2021; Liu et al., 2022). PPO and POD catalyze the oxidation of phenolic hydroxyl groups in flavonoids, thereby disrupting their structure and facilitating the degradation of flavonoids (Zhao et al., 2021; Pourcel et al., 2007). Differential expression of these hub genes might be another internal factor contributing to the reduction of flavonoid degradation induced by Fe. But effect of the two enzymes on the crosstalk between flavonoid and sugar metabolism still requires further investigation.

## 5 Conclusion

**Figure 7:**
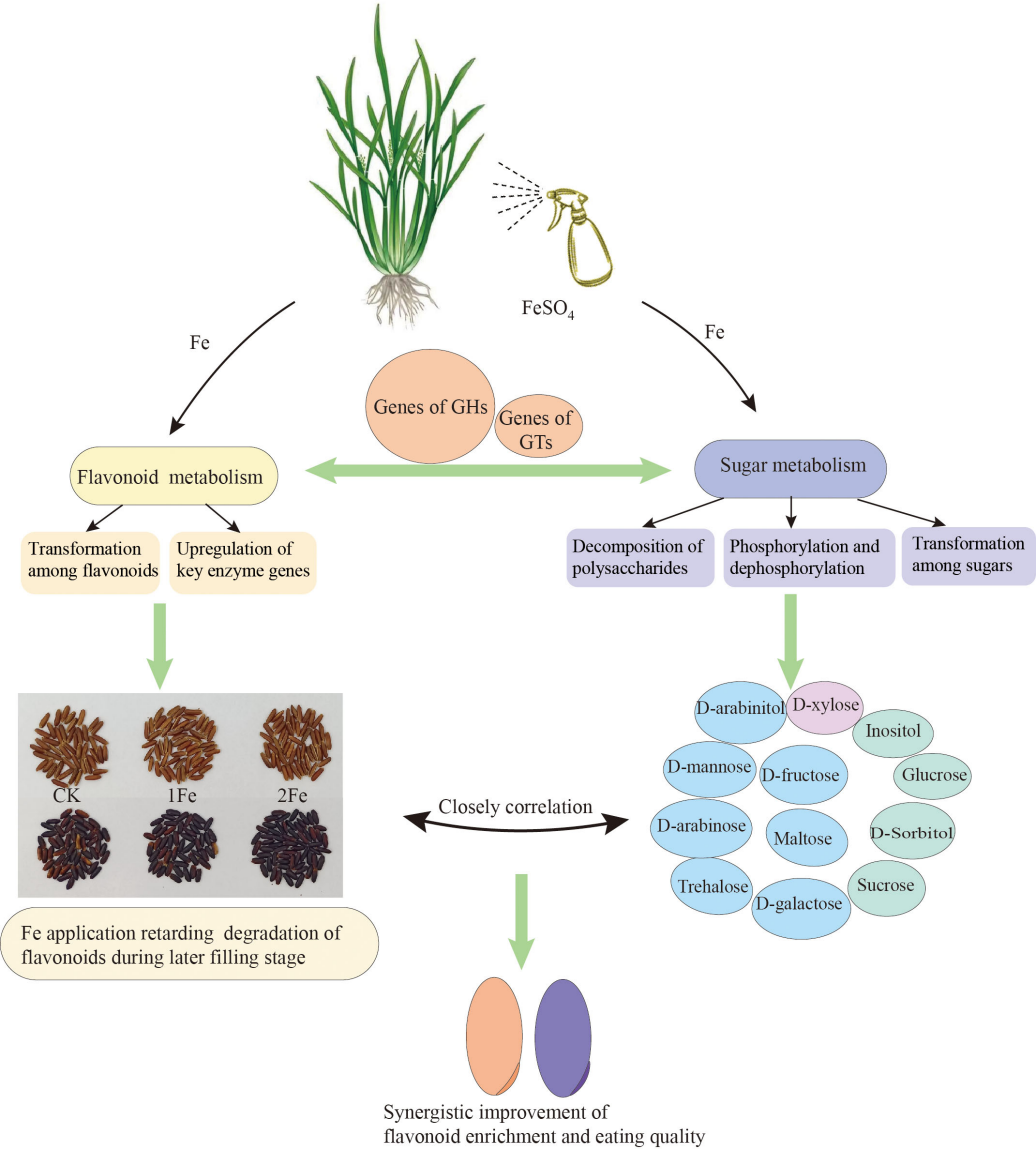
Schematic diagram illustrating the regulation of foliar application of iron on flavonoid and sugar metabolism in colored rice. Pink circle indicates that Fe treatment increases the content in both varieties. Green circles indicate that Fe treatment decreases the content in both varieties. Blue circles indicate that the effect of Fe treatment differs between the two varieties. BR, black rice Nanheinuo; RR, red rice Yuhongdao 5815; GHs, glycoside hydrolases; GTs, glycosyltransferases.

The expression of genes annotated as key enzymes in phenylpropanoid and flavonoid biosynthesis, including ANS, CHI, F3’5’H, F3’H, CHS, 4CL, DFR, F3H, and PAL, was significantly upregulated in BR under Fe treatment. Many DEGs were associated with flavonoid transformation. This suggests that Fe treatment may impact the transformation and storage forms of flavonoids during maturity, and delay flavonoid degradation in during the later filling stage. Additionally, foliar Fe application significantly influenced sugar and sugar alcohol content by regulating the expression of genes involved in polysaccharide decomposition, phosphorylation and dephosphorylation processes, and sugar interconversion. A close relationship was observed between sugar and flavonoid metabolism in the grains of colored rice, primarily mediated by the differential expression of hub genes annotated as GHs (**Figure 7**). These findings will support efforts to enhance flavonoid enrichment and improve the quality of colored rice.

## Data Availability

The data presented in the study are deposited in the Gene Expression Omnibus (GEO) repository (https://www.ncbi.nlm.nih.gov/geo/query/acc.cgi?acc=GSE326035), accession number: GSE326035.
