# Supplementary material for: Foliar iron application delays flavonoid degradation during the later grain-filling stage and regulates sugar metabolism in colored rice
Source: Front Plant Sci. 2026 Apr 7;17:1787767. doi: 10.3389/fpls.2026.1787767 (PMC13096702; doi:10.3389/fpls.2026.1787767)
Supplement: Supplementary file 1 [file DataSheet1.pdf]

Supplementary Material

Supplementary Table 1. Standard equations for sugars and sugar alcohols.

Compounds	Class	RT	Equation	R2
D-Xylose	monosaccharide	3.951	y = 0.651382 x - 8.769057E-005	0.9997
D-Arabinose	monosaccharide	4.023	y = 0.851245 x - 1.709393E-004	0.9996
Xylulose	monosaccharide	4.167	y = 1.126345 x + 0.002958	0.9989
D-Arabinitol	monosaccharide	4.594	y = 1.129333 x + 3.523394E-004	0.9959
1,5-Anhydroglucitol	monosaccharide	6.268	y = 0.702011 x - 1.650512E-004	0.9995
D-Fructose	monosaccharide	6.442	y = 0.757562 x - 2.883927E-005	0.9976
D-Mannose	monosaccharide	6.615	y = 0.526224 x - 3.860021E-005	0.9960
D-Galactose	monosaccharide	6.678	y = 0.410171 x - 2.076671E-004	0.9992
Glucose	monosaccharide	6.767	y = 0.582265 x - 5.612422E-005	0.9971
D-Sorbitol	monosaccharide	7.249	y = 2.481362 x + 1.850106E-004	0.9955
2-Acetamido-2-deoxy-D-glucopyranose	monosaccharide	9.129	y = 0.017262 x - 1.324349E-005	0.9938
Inositol	monosaccharide	9.237	y = 0.866938 x + 1.381460E-004	0.9942
Sucrose	disaccharide	14.400	y = 1.380380 x - 0.010214	0.9941
Cellobiose	disaccharide	14.922	y = 0.161946 x - 8.947886E-005	0.9900
Maltose	disaccharide	15.158	y = 0.174527 x - 7.215607E-005	0.9940
Trehalose	disaccharide	15.229	y = 1.621102 x - 0.001542	0.9966
Raffinose	trisaccharide	20.990	y = 0.181566 x - 1.687283E-004	0.9944
1, 6-dehydrated -β -D-glucose	monosaccharide	4.569	y = 0.226937 x - 5.300038E-005	0.9991

Supplementary Table 2 The primer sequences for qPCR

gene ID		primer (5'→3')
LOC4343325	F	GCGGGGATCATCGTCAACA
LOC4343325	R	AAGCAGAGGAACACGACCGA
LOC107276307	F	CGTCTCCCAACGACCTGT
LOC107276307	R	CCTTGCGTATCACCCACA
LOC4343353	F	CGCTTCCTGTGGGTGCTGC
LOC4343353	R	CCCCCTCCCCTTGGTCTTG
LOC4340373	F	TTCCCCTTCAGCTCCCAC
LOC4340373	R	GAACCTCATGTTCCCCGG
LOC4332719	F	ATGCTTGCATTCTCTGGTTG
LOC4332719	R	ATGTTGTTTCTCCGGGTGTT
LOC4341944	F	CGAAGAACCAGCCGATGA
LOC4341944	R	GCGGTAGGAGGCCGAGAA
LOC4333312	F	TCGTCATTGGTCATAGCGCA
LOC4333312	R	ATCCCACATGGACAAGGCTG
LOC4330775	F	CCTTGCCCCACTCAAAGATGT
LOC4330775	R	CGGGTTCGTCGTGTGTATGA
LOC4336505	F	AGGGGTGTTTACATGGAGGC
LOC4336505	R	AAGTGACCTGAAACCTGGCG
UBQ5-F	F	GACTACAACATCCAGAAGGAGTC
UBQ5-R	R	TCATCTAATAACCAGTTCGATTTC

Supplementary Table 3 Effect of Fe treatments on the content of sugars and sugar alcohols in grains of colored rice.

Compounds	Class	BR		RR	
		Control	1Fe	Control	1Fe
Glucose (mg/g)	monosaccharide	0.277±0.012a	0.183±0.008b	0.115±0.006c	0.086±0.002d
D-Fructose (μg/g)	monosaccharide	71.121±2.644c	74.399±4.138bc	92.746±2.148a	78.986±2.111b
D-Galactose (μg/g)	monosaccharide	18.413±0.996a	19.337±0.543a	6.345±0.063c	7.675±0.319b
D-Xylose (μg/g)	monosaccharide	1.925±0.083c	6.248±0.165a	2.481±0.321c	4.117±1.189b
2-Acetamido-2-deoxy-D-glucopyranose (μg/g)	monosaccharide	21.878±2.876a	21.253±2.583a	8.633±0.135b	8.773±0.299b
D-Mannose (μg/g)	monosaccharide	2.109±0.336a	1.439±0.274b	0.417±0.123c	0.279±0.139c
D-Arabinose (μg/g)	monosaccharide	5.805±0.166ab	6.051±0.234a	5.534±0.001b	3.748±0.360c
D-Sorbitol (μg/g)	sugar alcohol	17.117±0.777b	13.122±1.208c	30.144±1.357a	18.372±0.601b
1,5-Anhydroglucitol (μg/g)	sugar alcohol	32.814±2.813a	31.570±1.516a	90.051±1.668b	88.405±4.590b
D-Arabinitol (μg/g)	sugar alcohol	6.847±0.211a	5.420±1.063a	0.895±0.189c	2.820±0.716b
Inositol (μg/g)	sugar alcohol	43.156±1.825a	36.059±1.664b	19.962±1.230c	14.585±0.451d
Maltose (μg/g)	disaccharide	34.375±2.053a	18.513±1.866b	13.653±0.420c	17.037±0.080b
Trehalose (μg/g)	disaccharide	25.603±1.002a	17.357±0.642b	1.083±0.085d	2.729±0.291c
Sucrose (mg/g)	disaccharide	15.546±0.358a	13.918±0.493b	9.405±0.328c	8.161±0.254d
Raffinose (mg/g)	trisaccharide	3.816±0.172a	3.982±0.183a	0.417±0.028b	0.342±0.005b
D-Xylucose (μg/g)	monosaccharide	-	-	0.968±0.056a	0.913±0.196a
1, 6-dehydrated -β -D-glucose (μg/g)	monosaccharide	6.285±0.385a	6.197±0.190a	-	-
Cellobiose (μg/g)	disaccharide	6.285±0.385a	6.197±0.190a	-	-
Starch (%)	polysaccharide	65.578±1.973a	64.161±0.453a	77.642±0.625b	79.961±0.648b

Means (± SD) sharing the same letter within a row are not significantly different at the 0.05 significance level. '-': No substance was detected.

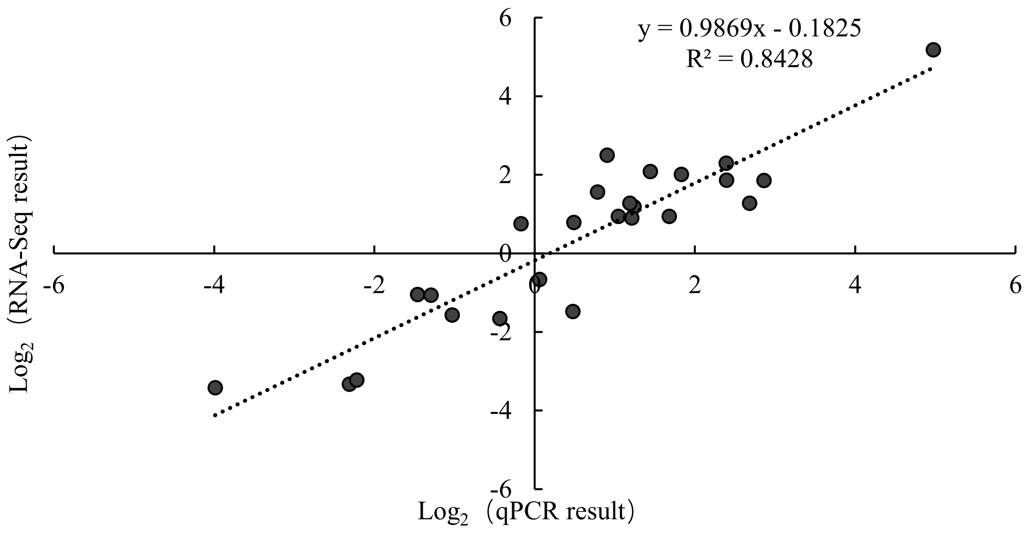

Supplementary Figure 1 The correlation between RNA-seq and qPCR results, qPCR result: $2^{\triangle\triangle Ct}$, RNA-seq result: FPKM (control)/ FPKM (iron treatment)

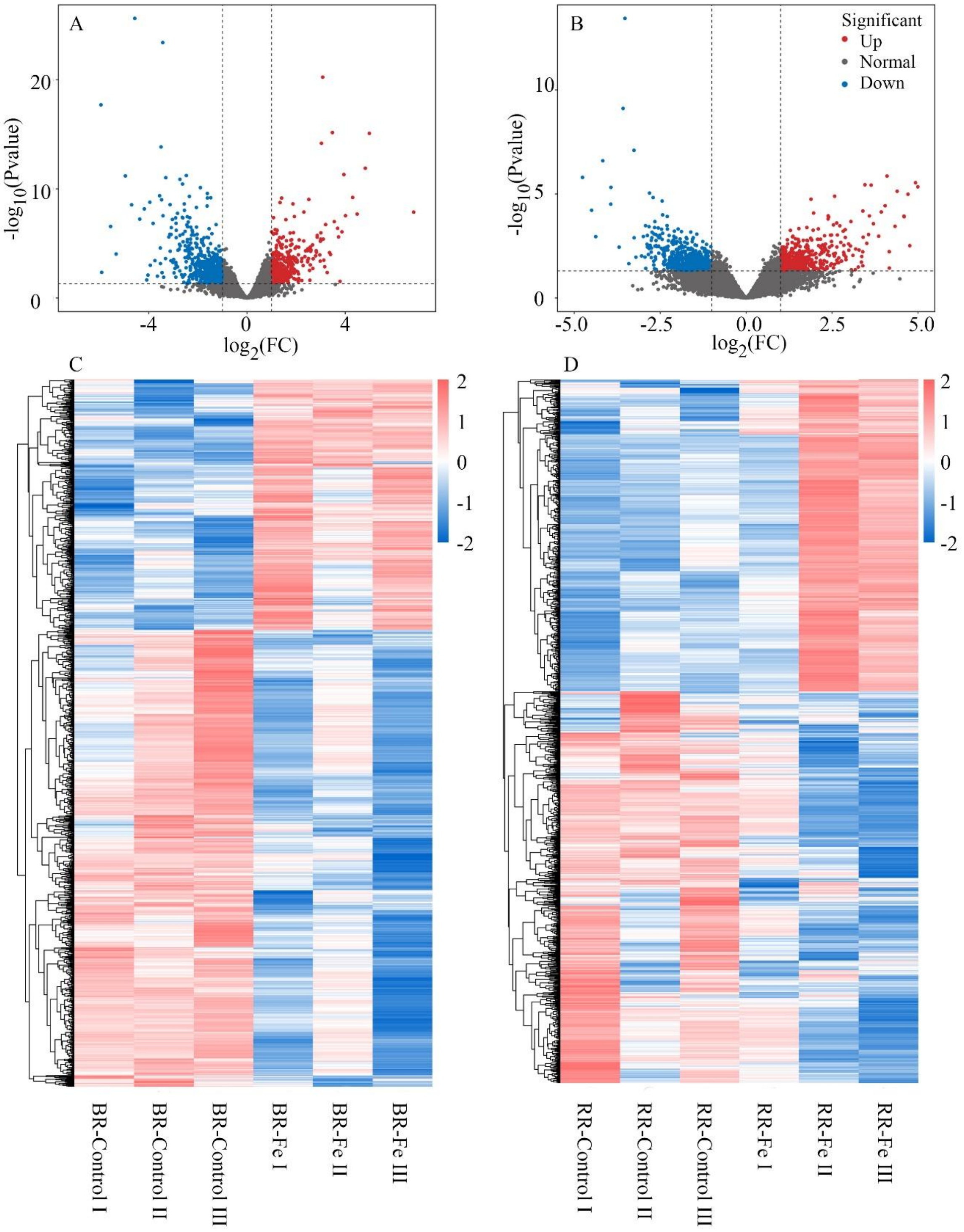

Supplementary Figure 2. Volcano plot and heatmap of significantly differentially expressed genes in black rice Nanheinuo (BR) (A, C) and red rice Yuhongdao 5815 (RR) (B, D). BR-Control: control treatment of BR; BR-Fe: Fe treatment of BR; RR-Control: control treatment of RR; RR-Fe: Fe treatment of RR.

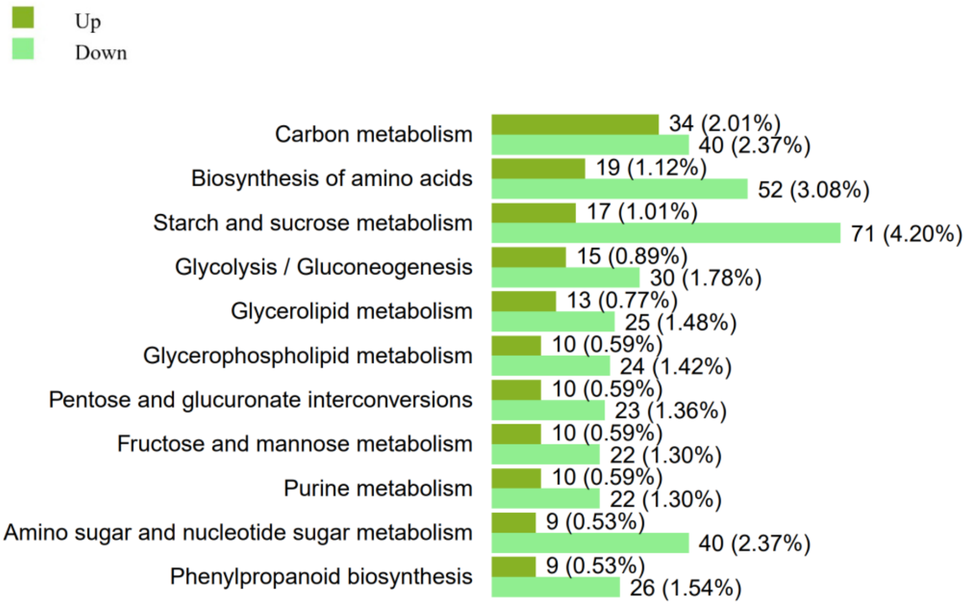

Supplementary Figure 3. Metabolic pathways of DEGs assigned to the KEGG database between grains at 20 days after flower and grains at 15 days after flower in Yuhongdao 5815 (RR). DEGs: significantly differentially expressed genes.
